# Hydroxyacyl-CoA dehydrogenase trifunctional multienzyme complex subunit beta gene as a tumour suppressor in stomach adenocarcinoma

**DOI:** 10.3389/fonc.2022.1069875

**Published:** 2022-11-23

**Authors:** Yun Li, Jian-Bo Xiong, Zhi-Gang Jie, Hui Xiong

**Affiliations:** ^1^ Department of Digestive Surgery, Digestive Disease Hospital, The First Affiliated Hospital of Nanchang University, Nanchang, Jiangxi, China; ^2^ Gastrointestinal Surgical Institute of Nanchang University, Nanchang, Jiangxi, China

**Keywords:** stomach adenocarcinoma, HADHB, KLF4, cell proliferation, Hippo-YAP pathway

## Abstract

**Background:**

Stomach adenocarcinoma (STAD) is the most common type of gastric cancer. In this study, the functions and potential mechanisms of hydroxyacyl-CoA dehydrogenase trifunctional multienzyme complex subunit beta (HADHB) in STAD were explored.

**Methods:**

Different bioinformatics analyses were performed to confirm HADHB expression in STAD. HADHB expression in STAD tissues and cells was also evaluated using western blot, qRT-PCR, and immunohistochemistry. Further, the viability, proliferation, colony formation, cell cycle determination, migration, and wound healing capacity were assessed, and the effects of HADHB on tumour growth, cell apoptosis, and proliferation in nude mice were determined. The upstream effector of HADHB was examined using bioinformatics analysis and dual luciferase reporter assay. GSEA was also employed for pathway enrichment analysis and the expression of Hippo-YAP pathway-related proteins was detected.

**Results:**

The expression of HADHB was found to be low in STAD tissues and cells. The upregulation of HADHB distinctly repressed the viability, proliferation, colony formation, cell cycle progression, migration, invasion, and wound healing of HGC27 cells, while knockdown of HADHB led to opposite effects. HADHB upregulation impeded tumour growth and cell proliferation, and enhanced apoptosis in nude mice. KLF4, whose expression was low in STAD, was identified as an upstream regulator of HADHB. KLF4 upregulation abolished the HADHB knockdown-induced tumour promoting effects in AGS cells. Further, HADHB regulates the Hippo-YAP pathway, which was validated using a pathway rescue assay. Low expression of KLF4 led to HADHB downregulation in STAD.

**Conclusion:**

HADHB might function as a tumour suppressor gene in STAD by regulation the Hippo-YAP pathway.

## Highlights

1. HADHB exhibited low expression in STAD tissues and cells
2. HADHB upregulation exhibited tumour suppressive effects in HGC27 and AGS cells
3. HADHB upregulation impeded tumour growth in xenograft BALB/c nude mice
4. Upregulation of KLF4 abolished the HADHB knockdown-induced tumour promoting effects in AGS cells
5. HADHB regulates the activation of the Hippo-YAP signalling pathway
6. YAP upregulation effectively reversed the HADHB upregulation-induced tumour suppressive effects in HGC27 cells

## Introduction

Gastric cancer (GC) is a common malignant tumour of the human digestive system and the third leading cause of cancer-associated deaths in the world (1). The mortality rate of GC patients is approximately 10% (2). GC can be classified into adenocarcinoma, signet ring-cell carcinoma, and undifferentiated carcinoma (3). However, stomach adenocarcinoma (STAD) is the most common pathological type and is characterized by easy metastasis (4, 5). STAD originates from the epithelial cells in the superficial layer of the stomach wall and is caused by malignant lesions of gastric gland cells (6). As early GC is usually asymptomatic, most patients are already in the advanced stage at the time of diagnosis and may even have distant metastasis at the time of diagnosis (7). Although multimodal therapies, such as surgery, target therapy, radiotherapy, and chemotherapy, have markedly improved in recent years, the five-year overall survival (OS) rate of patients with advanced GC is still less than 20% (8). Late diagnosis, lack of effective biomarkers, and treatment targets are the main reasons for STAD metastasis and recurrence. Thus, the identification of novel and prognostic biomarkers and effective treatment targets is essential for future treatment of STAD.

Hydroxyacyl-CoA dehydrogenase trifunctional multienzyme complex subunit beta (HADHB) is a type of fatty acid oxidase that can form mitochondrial trifunctional protein (MTP) with alpha (HADHA) (9). MTP is a multienzyme complex found in the inner membrane of the mitochondria, which can catalyse the last three steps of the beta-oxidation of fatty acids to provide energy for life activities (10, 11). Previous reports revealed that the fatty acid, ELOVL2, and upregulation of HADHB could increase beta-oxidation, which contributes to an increase in lipid utilization (12, 13). Notably, this alteration in lipid metabolism could provide biomass and energy for tumour progression (14). In recent years, extensive reports have demonstrated the tumour suppressing roles of HADHB in various cancers, such as colorectal cancer (CRC), oral squamous cell carcinoma (OSCC), and acute myeloid leukaemia (AML) (15–17). However, whether HADHB exerts the same effects in STAD has not been revealed.

In this study, we sought to determine the role of HADHB and its related molecular mechanisms in STAD. Based on our findings, the KLF4-HADHB axis could promote the progression of STAD by regulating the Hippo-YAP pathway, which indicates that HADHB might serve as a target for the treatment of STAD.

## Methods

### HADHB expression and survival analysis

In this study, the expression of HADHB in normal tissues and cancer tissues was analysed using UALCAN (http://ualcan.path.uab.edu/analysis-mir.html) and data from The Cancer Genome Atlas (TCGA) database. HADHB expression in STAD was evaluated using data collected from the Gene Expression Profiling Interactive Analysis (GEPIA) database (http://gepia.cancer-pku.cn/, tumour: 408 cases; no tumour: 211 cases). The overall survival (OS) of STAD patients was evaluated using Kaplan-Meier Plotter (http://kmplot.com/analysis/). In addition, we downloaded the TCGA-STAD data on Genomic Data Commons Data Portal (https://portal.gdc.cancer.gov/). Disease-free survival (DFS) and Cox risk analysis was conducted on these data using R software. The Kaplan-Meier method was also used to assess DFS. Thereafter, factors that were significant in the univariate survival analysis were substituted into the Cox multivariable survival analysis in the R package (18).

### Motif analysis and promotor binding site analysis

A Venn diagram analysis was performed to screen out the overlapping gene that co-exists among the three datasets, including HADHB transcription factor (TF), GC downregulated genes, and genes positively correlated with HADHB in TCGA. Motif analysis of KLF4 and the potential TF binding sites on the HADHB promotor was carried out using Jaspar database (https://jaspar.genereg.net/). KLF4 expression in STAD was analysed using data from GEPIA database (tumour: 408 cases; no tumour: 211 cases). The OS of STAD patients was further determined.

### Pathway enrichment analysis

Gene set enrichment analysis (GSEA) is widely used to explore potential signalling pathways involved in the regulation of specific target genes (19). In this study, GSEA, with HADHB as a target, was carried out using WebGestalt (http://www.webgestalt.org/) based on TCGA-STAD database. Wikipathway was used as the dataset in GSEA. The correlation between the identified hub genes that participated in the specific signalling pathway was further analysed by the cBioPortal platform (http://www.cbioportal.org/). A false discovery rate (FDR) < 0.05 and *P* < 0.05 were set as the cut-off values.

### Cell lines and clinical tissue collection

GES-1 cells and five STAD cell lines (HGC27, SNU-216, MKN-45, AGS, and FU97) were obtained from Procell Life Science&Technology Co.,Ltd. (Wuhan, China). GES-1, HGC-27, and SNU-216 cells were maintained in RPMI-1640 medium (#E600028), AGS cells were cultivated in Ham’s F12 medium (#E600014), and MKN-45 and FU97 cells were cultured in DMEM (#E600009). Before cell culture, 10% foetal bovine serum (FBS; #E600001) was added to the mediums. Cell culture was carried out in an incubator at 37°C with 5% CO_2_, and the culture medium was replaced twice per week (20). The reagents used for cell culture were provided by the BBI LIFE SCIENCES CORPORATION (Shanghai, China).

A total of 40 pairs of STAD tissues and their adjacent tissues were collected from patients diagnosed with STAD. After collection, one part of the tissue was immediately frozen in liquid nitrogen and stored at -80°C for a follow-up study, and the other part was fixed in formalin and embedded in paraffin for subsequent histological analysis (21). Informed consent was obtained from each participant. This study was approved by the Ethics Committee of The First Affiliated Hospital of Nanchang University (ID: IIT [2021] LLS No. 009, Nanchang, China).

### Western blot analysis

The relative protein expression levels of HADHB, metastasis-related factors, and signalling pathway-associated proteins in STAD tissues and GC cells were evaluated using western blot. The extraction of total tissue and cellular proteins was performed using RIPA lysis buffer (#C500005, Sangon Biotech, Shanghai, China) with a protease inhibitor cocktail (#P1005, Beyotime, Shanghai, China). Protein concentration was determined using a BCA kit (#PC0020, Solarbio, Beijing, China). Thereafter, western blot analysis was carried out. Briefly, the proteins of interest were separated using SDS-PAGE, transferred onto the PVDF membranes, and blocked in 5% BSA at room temperature (RT) for 1 h. Incubation with the primary antibody was performed overnight at 4°C followed by incubation with HRP-labelled secondary antibody for 1 h at RT. The membranes were rinsed with phosphate buffer saline (PBS) after each step. Finally, ECL reagent (#P0018S, Beyotime) was used for signal development (22). The results were obtained using LAS-3000 (FUJIFILM, Tokyo, Japan) system.

The following antibodies were provided by Proteintech (Wuhan, China): primary antibodies—anti-HADHB (29091-1-AP), anti-E-cadherin (20874-1-AP), anti-Vimentin (10366-1-AP), anti-N-cadherin (22018-1-AP), and Goat anti-Rabbit IgG secondary antibody—(SA00001-15). The following antibodies were provided by Abclonal (Wuhan, China): primary antibodies—anti-TEAD4 (A4151), anti-YAP (A1001) and anti-GAPDH (AC001).

### Immunohistochemistry analysis

HADHB and Ki67 expression in the prepared tumour tissues were assessed using IHC. Briefly, the prepared tissues were fixed in formalin, embedded in paraffin, and sliced into 3-μm thick sections. Thereafter, the sections were heated at 60°C for 2 h, dewaxed in xylene, and rehydrated in grade ethanol. Heating with citrate buffer was carried out for 30 min to induce antigen retrieval. The sections were then treated with 3% H_2_O_2_ for 15 min to block endogenous peroxidase. Incubation with primary antibodies, including HADHB (ab230667, Abcam) and Ki67 (ab15580, Abcam), was carried out overnight at 4°C followed by incubation with HRP-labelled secondary antibody for 1 h at 25°C. The sections were then stained with diaminobenzidine (DAB, #P0202, Beyotime) and the nucleus was counterstained with haematoxylin (#C0107, Beyotime). Membranes were incubated with PBS instead of the antibodies to serve as the negative control (NC) (21). The results were visualized and photographed using a microscope (Olympus, Japan).

### qRT-PCR analysis

The relative expression levels of HADHB, KLF4, and YAP in STAD tissues and cells were determined using qRT-PCR. Briefly, total RNA isolation was performed using TRIZOL reagent (#B610409, BBI LIGE SCIENCES CORPORATION). A spectrophotometer (HACH, Shanghai, China) was used to determine the quality and concentration of the isolated RNAs. Reverse transcription was then performed using the first strand cDNA synthesis kit (#k1612, Thermo Scientific) and the expression of target genes was determined using the BeyoFast™ SYBR Green One-Step qRT-PCR Kit (#D7277S, Beyotime, Shanghai, China). GAPDH was set as an internal control. The 2^-△△Ct^ method was used to calculate gene expression (23). The primer sequences used for qRT-PCR were as follows: HADHB, F, 5’- CTGAACCTTGCTCCGAGAGG-3’, R, 5’- TTTTGGTCTGGACAGCTGGG-3’; KLF4, F, 5’- CAGTCCCGGGGATTTGTAGC-3’, R, 5’- GAAGAAGGTGGGGTGAGCAT-3’; YAP, F, 5’- GAACTCGGCTTCAGGTCCTC-3’, R, 5’- GGTTCATGGCAAAACGAGGG-3’; and GAPDH, F, 5’- CCATGGGGAAGGTGAAGGTC -3’, R, 5’- TCGCCCCACTTGATTTTGGA -3’.

### Cell transfection

The small-interfering (si)RNAs of HADHB, siRNA1-HADHB (Sense, GGUCUCUGUUGUCACUAAAGA; Anti-sense, UUUAGUGACAACAGAGACCAA) and siRNA2-HADHB (Sense, GAAUGACUAUCUUGACUUACC; Anti-sense, UAAGUCAAGAUAGUCAUUCUG), si-NC (Sense, UUCUCCGAACGUGUCACGUTT; Anti-sense, ACGUGACACGUUCGGAGAATT), and the overexpressing plasmids (pcDNA3.1-HADHB, pcDNA3.1-KLF4, and pcDNA3.1-YAP) were synthesized by Tsingke Biotechnology Co., Ltd (Beijing, China). Cell transfection was carried out using Lipofectamine™ 2000 reagent (#52887, Invitrogen, California, USA). HGC27 and AGS cells resuspended in serum-free medium were respectively seeded in 6-well plates (5 × 10^5^/ml, 1 ml per well), which were cultured overnight in a humidified incubator at 37°C with 5% CO_2_. The synthesized siRNA or recombined plasmids were mixed gently with serum-free medium followed by Lipofectamine™ 2000 and maintained at RT for 20 min. The mixture was added to the cell culture and maintained under the same condition for 4 h. Finally, the culture medium was discarded, and complete medium was added. Cell collection was carried out 48 h post-transfection for subsequent experiments (24).

### Cell viability analysis

Cell viability was determined using the CCK-8 assay (#C0037, Beyotime). After transfection, HGC27 and AGS cells were seeded in 96-well plates (1 × 10^5^/ml, 100 μl per well), respectively, and cultured in a humidified incubator at 37°C with 5% CO_2_ for different times (0 h, 24 h, 48 h, 72 h, and 96 h). The culture medium was then discarded, and 100 μl of CCK-8 reagent was added. The cells were cultured for another 1 h (25) and the optical density (OD) value was measured using a microplate reader (Bio-Rad, Hercules, CA).

### Cell proliferation analysis

Cell proliferation was measured using the EdU incorporation assay (#C00003, RIBOBIO, Guangzhou, China) and colony formation assay. For the EdU incorporation assay, the transfected HGC27 and AGS cells were seeded in 96-well plates (1 × 10^4^/ml, 100 μl per well) and cultured to normal growth stage at 37°C with 5% CO_2_. The culture medium was replaced with 100 μl of EdU reagent (50 mM) and cultured for another 2 h. After rinsing, cells were fixed in 50 μl of 4% paraformaldehyde (PFA, #P0099, Beyotime) at RT for 30 min and stained with 100 μl of 1 × Apollo solution at RT for 30 min in the dark (26). The nuclei were stained with DAPI reagent (#C1002, Beyotime) and the results were observed using a microscope.

For the colony formation assay, 200 μl of the transfected HGC27 and AGS cells and 100 μl of cell culture medium were added to a 35-mm petri dish and cultured in a humidified incubator at 37°C with 5% CO_2_ for 2-3 weeks until visible clones appeared. After washing with PBS, the cells were fixed in 1 ml of 4% PFA for 20 min, and then stained with 0.5% crystal violet (#C0121, Beyotime) for 10 min. Finally, the clones were captured and counted under a microscope.

### Cell cycle analysis

After transfection, HGC27 and AGS cells were collected in a tube (approximately 5 × 10^4^ cells) and centrifuged at 300 × g for 10 min. The cells were then suspended in 1 ml of pre-cooled PBS and re-centrifuged. The supernatant was discarded, and the cells were suspended in the remaining PBS, fixed in pre-cooled absolute ethyl alcohol at -20°C for 1 h, washed, centrifuged at 300 × g for 10 min, and suspended in 100 μl of RNase A (#ST579, Beyotime). The mixture was then incubated in a 37°C water bath for 30 min. After the addition of propidium iodide (PI, # ST511, Beyotime), the mixture was maintained at 4°C for 30 min in the dark (27). Finally, cell cycle determination was conducted using the FACScan flow cytometer (BD Biosciences, NJ, USA). DNA content was determined using the CellQuest software (BD Biosciences).

### Cell migration and invasion assessment

Cell migration was assessed using the transwell migration assay and cell scratch assay. For the migration assay, 100 μl of the transfected HGC27 and AGS cells suspended in serum-free medium was respectively seeded in the upper chamber (2 × 10^5^/ml). Meanwhile, the lowered chamber was supplied with 700 μl of complete medium containing 10% FBS. The chambers were maintained in an incubator at 37°C with 5% CO_2_ for 48 h. Thereafter, the cells that did not migrate were gently removed *via* wiping. After rinsing with PBS, the chambers were fixed in 4% PFA for 20 min, and then stained with 0.2% crystal violet reagent for 10 min. The film was then removed from the chamber, dried, and sealed with neutral resin (#G8590, Solarbio). The results were observed and captured with a microscope (28). Finally, 6 fields were randomly selected, and cell counting was conducted using the IPP software.

In the invasion assay, the upper chamber used was pre-coated with 100 μl of matrigel matrix (Corning, USA). The remaining procedure was the same as that of the migration assay.

For the cell scratch assay, the transfected HGC27 and AGS cells were respectively seeded in 6-well plates (5 × 10^5^/ml, 1 ml per well), and cultured at 37°C with 5% CO_2_ for 48 h. A 200-μl pipette was used to remove cells and create scratches. After washing, cells were maintained in serum-free medium for another 48 h (28). Finally, the results were observed and photographed with a microscope. The wound healing rates were further analysed using IPP software.

### Construction of the xenograft STAD model in nude mice

Eight BALB/c nude mice (aged 4-6 weeks) were purchased from Jinan Pengyue experimental animal breeding Co., Ltd (Jinan, China) and reared at 25-27°C with humidity of 45%-50% under standard 12 h light/dark cycle conditions for one week. During this period, mice were randomly divided into 2 two groups (4 in each group) and granted access to food and water. HGC27 cells transfected with empty pcDNA3.1 or pcDNA3.1-HADHB plasmids were collected at the logarithmic growth phase and used to create a single-cell suspension (3 × 10^7^/ml) with serum-free RPMI-1640 medium. Thereafter, 0.2 ml of the prepared cell suspension was subcutaneously inoculated into the dorsal flanks of nude mice. After rearing under the same conditions for ten days, tumour size (width and length) was measured every five days until the rearing time reached 30 days. Thereafter, tumour volume was calculated based on the data recorded, and a tumour growth curve was generated. The formula used to calculate tumour volume was as follows: tumour volume (V, mm^3^) = 0.5 × length × width × width. Mice were exsanguinated on day 30, and their tumour tissues were collected, weighed, and fixed in 4% PFA for follow-up analysis (29). The animal experimental protocol was performed in accordance with the Guide for the Care and Use of Laboratory Animals and was approved by The First Affiliated Hospital of Nanchang University (ID: SD [2021] DLS No. 011).

### Histopathological analysis

Histopathological analysis of the collected tumour tissues was carried out using the Haematoxylin and Eosin (H&E) Staining Kit (#C0105S, Beyotime) according to the product instructions. Briefly, the tumour tissues were successively fixed in 4% PFA, dehydrated using gradient ethanol, embedded in paraffin, and sliced into 4 μm sections. The sections were then deparaffinized in xylene, hydrated in gradient ethanol, stained with haematoxylin for 2 min and eosin for 1 min. Finally, the sections were sealed with neutral resin. The results were observed and photographed with a microscope (30).

### Cell apoptosis analysis

Apoptosis of the tumour tissue cells was analysed using One Step TUNEL Apoptosis Assay Kit (#C1086, Beyotime) according to the product instructions. In brief, the sections (4 μm) were deparaffinized in xylene, hydrated with gradient ethanol, and treated with DNase-free proteinase K (diluted with 10mM Tris-HCl pH7.4-7.8) at 37°C for 15 min. After washing, sections were incubated with 50 μl of TUNEL reagent at 37°C for 60 min in the dark (31). Finally, the sections were sealed with Antifade Mounting Medium (#P0126, Beyotime), and the results were observed and captured with a microscope.

### Dual-luciferase reporter assay

The interaction between HADHB and KLF4 was analysed using the Dual-luciferase reporter assay. The HADHB-wild type (WT) and HADHB-mutant type (MUT) reporter vectors were constructed by inserting the cDNA fragments of HADHB-WT (ACACACCCACA) and HADHB-MUT (GCACCCTCACA) into pGL3 vectors (#E1761, Promega, USA), respectively (32). HEK293T cells were seeded in 24-well plates (3 × 10^5^ cells per well) and maintained in DMEM supplemented with 10% FBS at 37 °C with 5% CO_2_ until 70-80% confluency was achieved. Thereafter, the HADHB-WT or HADHB-MUT or the empty pGL3 vectors (negative control, NC) were co-transfected with pcDNA3.1-KLF4 plasmids into HEK293T cells using Lipofectamine^TM^ 2000 reagent. After 48 h, the relative luciferase activity was detected using the Dual-Luciferase Reporter Assay System (#E1910, Promega, USA). The results were expressed as Renilla luciferase signal/Firefly luciferase signal (R/F).

### Statistical analysis

Three replicates were used in each experiment. GraphPad Prism software (GraphPad Software, USA) was used for statistical analysis. Data are expressed as mean ± standard deviation (SD). The variables were compared using Student’s *t*-test and One-way ANOVA. Correlation analysis of HADHB and KLF4 expression was performed using Pearson correlation analysis. *P* < 0.05 indicates statistical significance.

## Results

In the present study, HADHB was demonstrated to be down-regulated in STAD tissues and cells. Further, the KLF4-HADHB axis could promote the progression of STAD by regulating the Hippo-YAP pathway.

### The expression of HADHB is low in STAD tissues and cells

**Figure 1:**
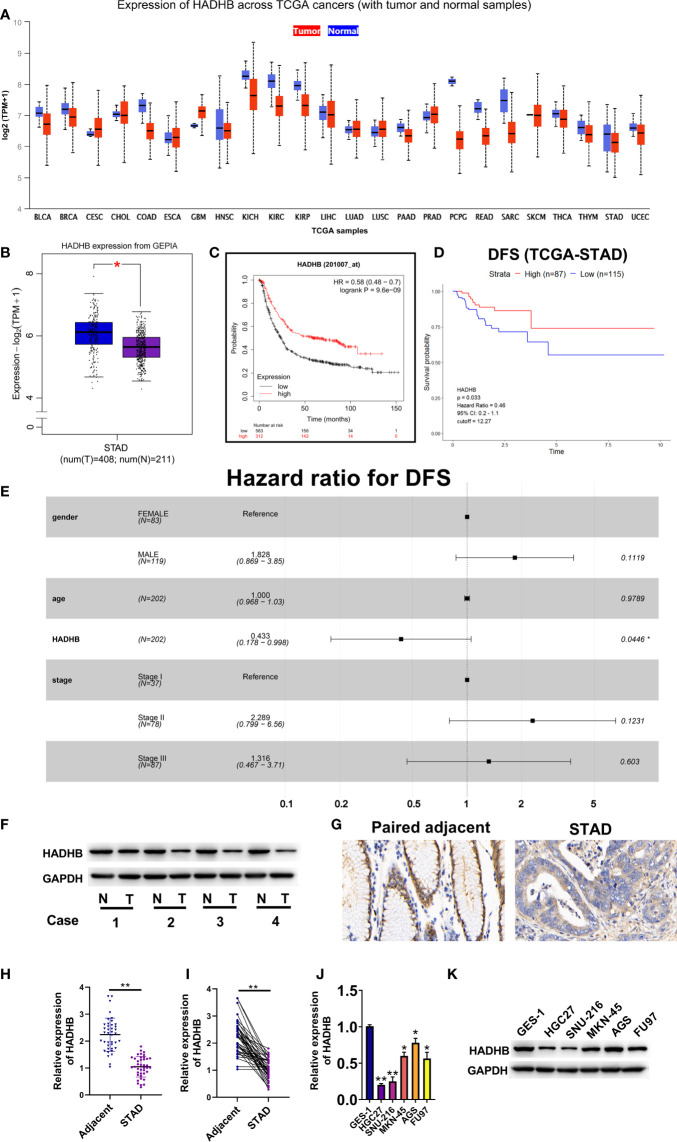
The expression of HADHB was low in STAD tissues and cell lines **(A)**. Differential mRNA expression of HADHB between normal tissues and tumour tissues in TCGA database. **(B)**. The mRNA expression of HADHB between normal tissues and tumour tissues in GEPIA. **(C)**. The relationship between the HADHB gene and overall survival (cutoff = 12.19) of STAD patients using Kaplan-Meier Plotter webtool. **(D)**. The relationship between the HADHB gene and disease-free survival (cutoff = 12.27) of TCGA-STAD patients using R software. **(E)**. Cox risk multivariable analysis of STAD. **(F)**. HADHB expression was determined using 4 pairs of significantly different clinical samples *via* western blot. **(G)**. HADHB expression was assessed *via* immunohistochemistry. **(H, I)**. HADHB expression in 40 pairs of clinical STAD tissues was determined using qRT-PCR. **(J, K)**. HADHB expression in STAD cell lines was determined using qRT-PCR and western blot. ^*^ *P* < 0.05, ^**^ *P* < 0.01.

HADHB is known as a tumour suppressor gene in various cancers (15, 33). In this study, we analysed the expression of HADHB in 24 tumours using UALCAN and data obtained from TCGA database. HADHB was found to have a low expression in various cancers, including STAD (**Figure 1A**). As the sample size of STAD included in TCGA database was limited, we opted to further analyse HADHB expression in STAD using the GEPIA database. The median expression level of HADHB in STAD tissues was notably lower than that in normal tissues (**Figure 1B**, *P* < 0.05). Based on OS (cutoff = 12.19) and DFS (cutoff = 12.27) analysis, low expression of HADHB is closely associated with the poor prognosis of STAD patients (**Figures 1C, D**). Furthermore, Cox risk analysis revealed that as HADHB expression decreased, the patient’s risk of death increased by 56.7%. Of note, other parameters (sex, age, and stage) were not identified as risk factors (**Figure 1E**). Based on these results, 4 pairs of significantly different clinical samples were selected to detect the expression of HADHB at the protein level and 40 pairs of clinical tissues were selected to detect its expression at the mRNA level. HADHB expression in STAD tissues was found to be markedly decreased relative to that found in adjacent normal tissues at the protein level and mRNA level (**Figures 1F, G, H, I**, both *P* < 0.01). The expression of HADHB in five STAD cell lines was also evaluated. Based on the results, HADHB expression in STAD cells was remarkably lower than that in GES-1 cells (**Figures 1J, K**, *P* < 0.05 or *P* < 0.01). Based on the results of qRT-PCR and western blot, HGC27 cells were selected for HADHB upregulation and AGS cells were selected for HADHB knockdown; HADHB expression was the lowest and highest in these cells, respectively.

### HADHB upregulation induces tumour suppressive effects in HGC27 and AGS cells

**Figure 2:**
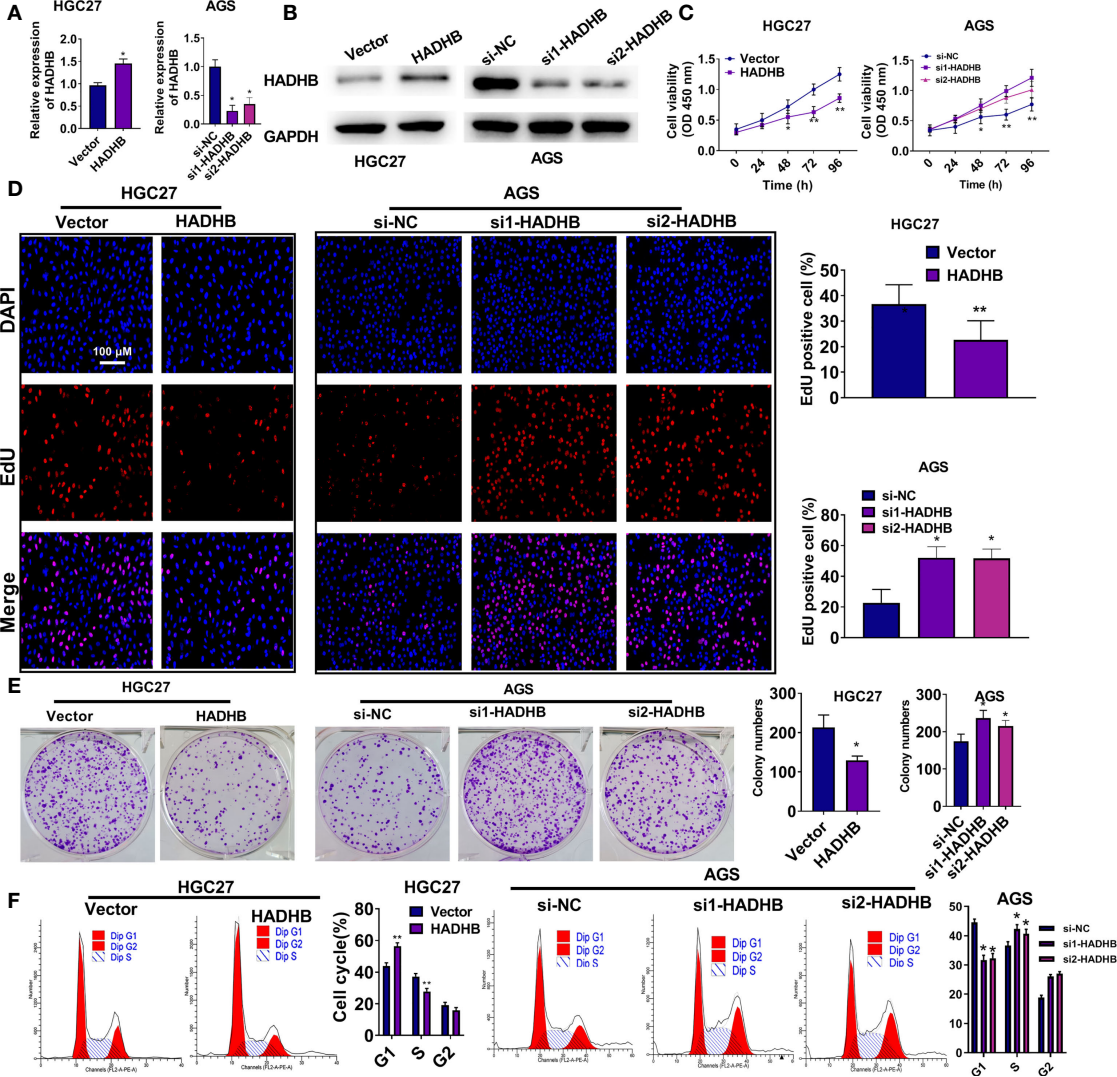
HADHB upregulation repressed the proliferation and cell cycle progression of HGC27 and AGS cells **(A, B)**. HADHB expression in the transfected HGC27 and AGS cells was determined using qRT-PCR and western blot analysis. **(C–F)**. The viability, proliferation, colony formation, and cell cycle of the transfected AGS and HGC27 cells were determined using the CCK-8 assay, EdU assay, colony formation assay, and flow cytometry analysis, respectively. ^*^ *P* < 0.05, ^**^ *P* < 0.01.

**Figure 3:**
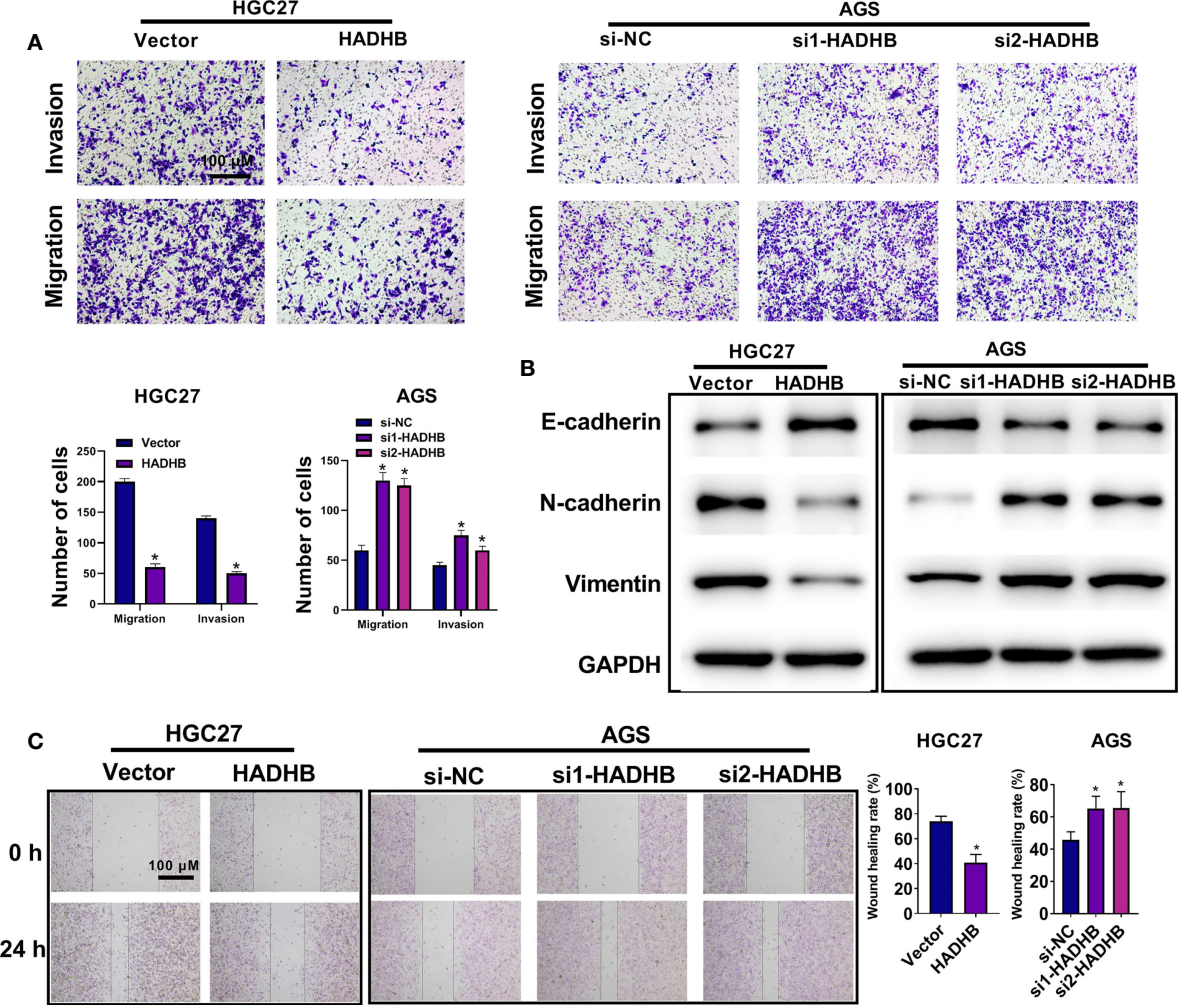
HADHB upregulation impeded the migration, invasion, and wound healing of HGC27 and AGS cells **(A)**. The invasion and migration of the transfected HGC27 and AGS cells were determined using the transwell assay. **(B)**. The expression of metastasis-related factors (Vimentin, N-cadherin, and E-cadherin) was evaluated using western blot analysis. **(C)**. Cell migration capacity of the transfected AGS and HGC27 cells was assessed using the wound healing assay. ^*^ *P* < 0.05.

To reveal the roles of HADHB in STAD progression, HADHB expression was upregulated and knocked down in HGC27 and AGS cells. **Figures 2A, B** shows that HADHB was successfully upregulated in HGC27 cells and knocked down in AGS cells (all *P* < 0.05). Of note, the silencing effect of siRNA1-HADHB was more significant than that of siRNA2-HADHB. Subsequent experiments revealed that HADHB upregulation notably repressed the viability of HGC27 cells as time progressed (**Figure 2C**, *P* < 0.05 or *P* < 0.01). HADHB upregulation also markedly decreased the ratios of EdU positive cells (**Figure 2D**, *P* < 0.05), restrained colony formation (**Figure 2E**, *P* < 0.05), distinctly increased the number of cells in G1 phase, and reduced the number of cells in S phase (**Figure 2F**, both *P* < 0.01). Such findings indicate that HADHB upregulation remarkably restrained cell cycle progression. The transwell assay revealed that cell migration and invasion were notably repressed by HADHB upregulation (**Figure 3A**, both *P* < 0.05). These results were confirmed by the elevated expression of metastasis-related E-cadherin and the reduced expression of Vimentin and N-cadherin (**Figure 3B**), and the decrease in wound healing rates (**Figure 3C**, *P* < 0.05). Interestingly, the effect of HADHB knockdown in AGS cells opposed that of HADHB upregulation in HGC27 cells (**Figures 2C, F**, **3A–C**, *P* < 0.05 or *P* < 0.01). These results imply that HADHB acts as a tumour suppressor gene *in vitro*, as its upregulation restrained the migration, invasion and proliferation of HGC27 and AGS cells.

### HADHB upregulation impedes tumour growth in xenograft BALB/c nude mice

**Figure 4:**
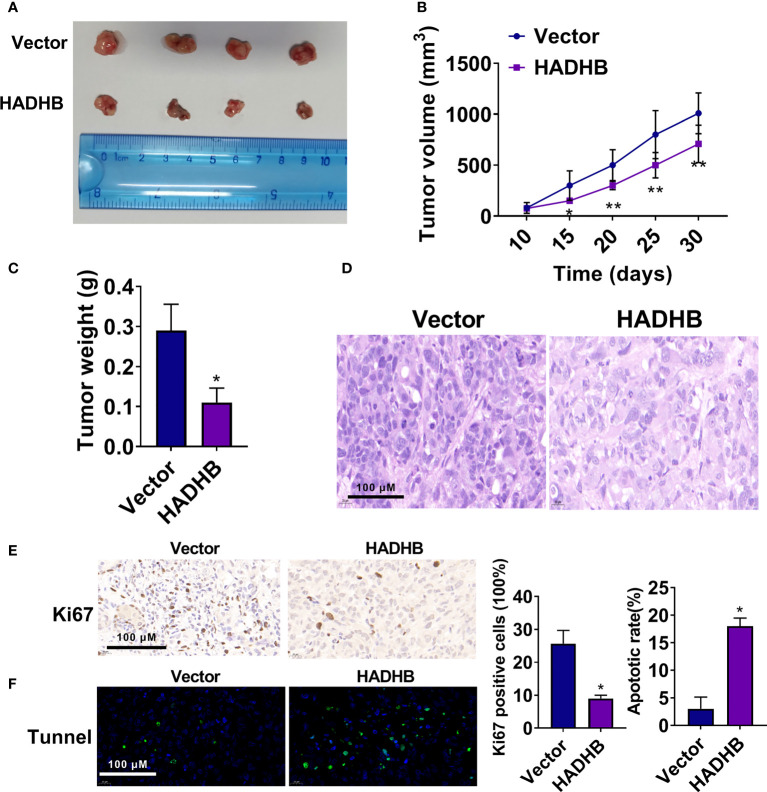
HADHB upregulation impeded tumour growth in xenograft BALB/c nude mice **(A)**. Images of the tumours (4 in each group). **(B)**. Tumour volume was calculated using the formula: tumour volume (V, mm^3^) = 0.5 × length × width × width. **(C)**. Tumour weight was determined. **(D)**. Histopathological analysis of the collected tumour tissues was performed using H&E staining. **(E, F)**. Tumour cell proliferation and apoptosis were assessed *via* immunohistochemistry with the Ki67 antibody and TUNEL assay, respectively. ^*^ *P* < 0.05, ^**^ *P* < 0.01.

As the above results proved the anti-tumour effects of HADHB *in vitro*, we carried out an *in vivo* assay to corroborate the results. **Figures 4A, B** show that tumour volume was markedly reduced by HADHB upregulation after day 15 compared with the control (*P* < 0.05 or *P* < 0.01), and the difference increased as time progressed. Tumour weight was also remarkably decreased by HADHB upregulation (**Figure 4C**, *P* < 0.05). H&E staining and IHC assay revealed that the tumour cell number and ratio of Ki67 positive cells were notably reduced by HADHB upregulation (**Figure 4D** and 4E, *P* < 0.05), indicating that cell proliferation in the generated tumours was notably repressed. The results of the TUNEL assay proved that cell apoptosis was markedly increased (**Figure 4F**, *P* < 0.05). Such findings indicate that HADHB upregulation impeded tumour growth in xenograft BALB/c nude mice by inhibiting cell proliferation and enhancing cell apoptosis.

### Upregulation of KLF4 abolishes the HADHB knockdown-induced tumour promoting effects in AGS cells

**Figure 5:**
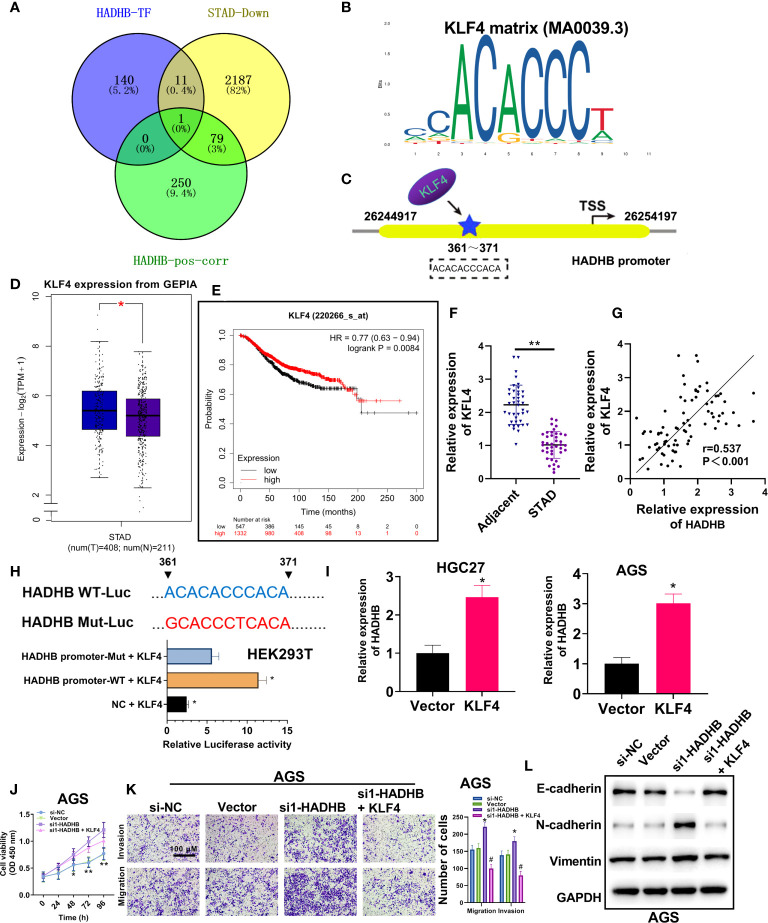
Upregulation of KLF4 abolished the HADHB knockdown-induced tumour promoting effects in AGS cells **(A)**. Venn diagram was generated to screen out the overlapping gene among the three datasets, namely HADHB transcription factor (TF), gastric cancer downregulated genes, and genes positively correlated with HADHB in TCGA. **(B, C)**. Motif analysis of KLF4 and HADHB promotor binding site analysis was conducted using Jaspar database. **(D)**. The mRNA expression of KLF4 between normal tissues and tumour tissues in GEPIA. **(E)**. Overall survival probability of STAD patients was analysed using Kaplan-Meier Plotter (cutoff = 9.01). **(F)**. KLF4 expression in 40 pairs of clinical STAD tissues was determined using qRT-PCR. **(G)**. The correlation between HADHB and KLF4 expression in STAD was derived using Pearson correlation analysis. **(H)**. The regulating relationship between HADHB and KLF4 was determined using the dual luciferase reporter assay. **(I)**. HADHB expression in KLF4-overexpressed AGS and HGC27 cells was determined using qRT-PCR. **(J, K)**. **(L)**. The expression of metastasis-related factors (Vimentin, N-cadherin, and E-cadherin) was evaluated using western blot analysis. The viability, migration, and invasion of the transfected AGS cells were determined using CCK-8 assay and transwell assay, respectively. ^*^ *P* < 0.05, ^**^ *P* < 0.01.

To determine the upstream regulatory mechanisms of HADHB in STAD, various bioinformatic analysis were carried out. Venn diagram revealed KLF4 as the only overlapping gene among the three datasets, namely HADHB TF, GC downregulated genes, and genes positively correlated with HADHB in TCGA (**Figure 5A**). The motif of KLF4 is displayed in **Figure 5B**. Of note, the potential binding site of KLF4 on the HADHB promotor is located at 361-371 bases (**Figure 5C**). KLF4 expression in STAD was determined using the GEPIA database. The median expression level of KLF4 was distinctly lower in STAD tissues than normal tissues (**Figure 5D**, *P* < 0.05). Besides, OS analysis revealed that the low expression of KLF4 was closely associated with the poor prognosis of STAD patients (**Figure 5E**, cutoff = 9.01). Collectively, these results indicate that the expression of KLF4 is low in STAD, which can lead to the downregulation of HADHB by binding to the 361-371 site of the HADHB promoter. To address this assumption, various experimental approaches were employed. qRT-PCR revealed that the expression of KLF4 was markedly reduced in STAD tissues (**Figure 5F**, *P* < 0.01). Further, KLF4 expression was found to be positively correlated with HADHB in STAD (**Figure 5G**, *P* < 0.01). The dual-luciferase reporter assay proved that the co-transfection of HADHB-WT and pcDNA3.1-KLF4 notably augmented luciferase activity compared with the co-transfection of HADHB-MUT and pcDNA3.1-KLF4 (**Figure 5H**, *P* < 0.05). These findings indicate that KLF4 could be an upstream effector of HADHB. KLF4 overexpression was performed in HGC27 and AGS cells. HADHB expression was found to be markedly elevated in the two cell lines (**Figure 5I**, both *P* < 0.05). siRNA1-HADHB and pcDNA3.1-KLF4 were subsequently co-transfected into AGS cells to validate the role of KLF4 in STAD. The upregulation of KLF4 distinctly relieved the HADHB knockdown-induced tumour promoting effects on cell viability (**Figure 5J**, P < 0.05 or P < 0.01), invasion, and migration (**Figures 5K, L**, both *P* < 0.05). Collectively, these findings imply that KLF4 might play a tumour suppressing role in STAD by interacting with the HADHB promoter at the 361-371 site to regulate its expression.

### HADHB regulates the Hippo-YAP signalling pathway

**Figure 6:**
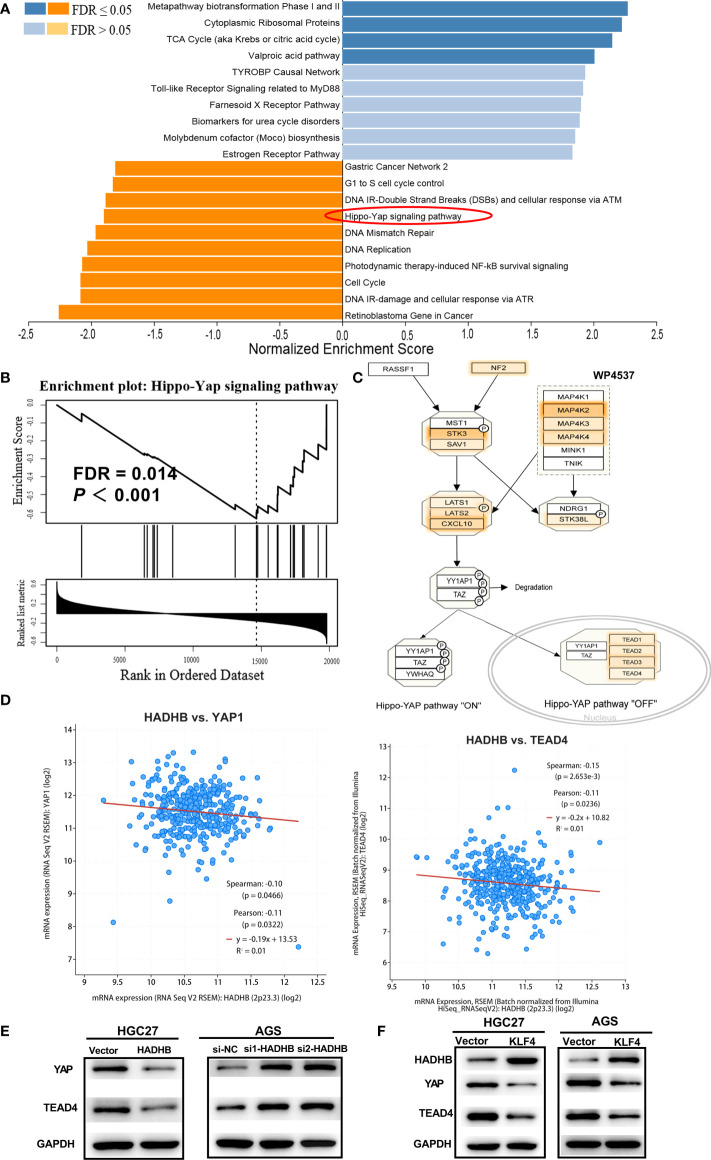
HADHB upregulation restrained the regulation of the Hippo-YAP signalling pathway **(A)**. Pathway enrichment analysis was carried out using GSEA and WebGestalt based on TCGA-STAD database. **(B)**. Enrichment plot of the Hippo-Yap signalling pathway. **(C)**. Pathway view of the Hippo-YAP signalling pathway, with the leading-edge protein highlighted. **(D)**. Analysis of the correlation between HADHB and YAP, as well as HADHB and TEAD4 was performed using the cBioPortal platform. **((E–F))**. The expression of HADHB and the Hippo-YAP signalling pathway-related factors (YAP and TEAD4) in the transfected AGS and HGC27 cells was evaluated *via* western blot.

To reveal the underlying mechanisms of HADHB in STAD, pathway enrichment analysis was carried out using GSEA. The 14 most significantly enriched signalling pathways were Cytoplasmic Ribosomal Proteins, Metapathway biotransformation Phase I and II, TCA Cycle, Valproic acid pathway, Gastric Cancer Network 2, G1 to S cell cycle control, DNA IR-Double Strand Breaks (DSBs), and cellular response *via* ATM, DNA Mismatch Repair, Hippo-YAP signalling pathway, DNA Replication, DNA IR-damage and cellular response *via* ATR, Photodynamic therapy-induced NF-kB survival signalling, and Cell cycle and Retinoblastoma Gene in Cancer (**Figure 6A**, FDR < 0.05). Of these pathways, the first 4 pathways were found to be activated by HADHB expression, while the last 10 pathways were inhibited by HADHB expression. As the Hippo-YAP signalling pathway is the most widely reported pathway in GC (34–36), this pathway was selected for further investigation. The corresponding pathway enrichment plot is shown in **Figure 6B**, and the pathway view is displayed in **Figure 6C**. As YAP is a crucial protein involved in the Hippo-YAP signalling pathway (37, 38), its expression was also detected in the follow-up investigation. The correlation between HADHB and YAP, as well as HADHB and TEAD4 were derived using the cBioPortal platform. YAP and TEAD4 were found to be negatively correlated with HADHB expression (**Figure 6D**). Thereafter, the expression levels of YAP and TEAD4 in the transfected HGC27 and AGS cells were measured. HADHB upregulation markedly reduced the levels of YAP and TEAD4,. HADHB knockdown led to the opposite effects. These results indicate that HADHB upregulation might induce tumour suppressive effects in HGC27 and AGS cells by regulating the Hippo-YAP signalling pathway. Further, the overexpression of KLF4 was found to inhibit the protein expression of HADHB, followed by YAP and TEAD4 (**Figure 6F**).

### YAP upregulation effectively reverses the HADHB upregulation-induced tumour suppressive effects in HGC27 cells

**Figure 7:**
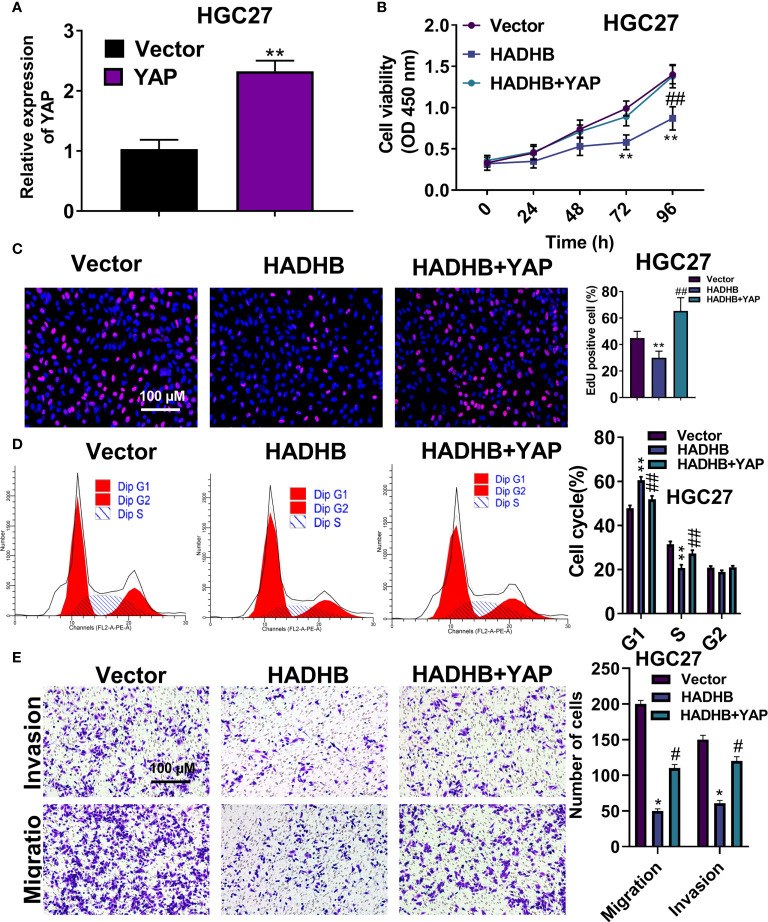
YAP upregulation effectively reversed the HADHB upregulation-induced tumour suppressing effects in HGC27 cells **(A)**. YAP expression in pcDNA3.1-YAP transfected HGC27 cells was measured using qRT-PCR. **(B–E)**. The viability, proliferation, cell cycle progression, migration, and invasion of the transfected HGC27 cells were determined using the CCK-8 assay, EdU assay, flow cytometry analysis, and transwell assay, respectively. ^*^ *P* < 0.05, ^**^ *P* < 0.01, ^#^ *P* < 0.05, ^##^ *P* < 0.01.

To verify the roles of the Hippo-YAP signalling pathway in STAD, we performed pathway rescue assay by co-transfecting pcDNA3.1-HADHB and pcDNA3.1-YAP plasmids into HGC27 cells. **Figure 7A** shows that YAP was successfully overexpressed in HGC27 cells (*P* < 0.01). Subsequent experiments revealed that YAP upregulation markedly abolished the HADHB upregulation-induced repressive effects on cell viability (**Figure 7B**, all *P* < 0.01), proliferation (**Figure 7C**, *P* < 0.01), cell cycle progression (**Figure 7D**, both *P* < 0.01), and migration and invasion (**Figure 7E**, both *P* < 0.05). These results suggest that the HADHB upregulation-induced tumour suppressive effects in HGC27 cells by regulating the Hippo-YAP signalling pathway.

## Discussion

In this study, the expression of HADHB was found to be low in STAD tissues. HADHB upregulation notably restrained the viability, proliferation, and migration of HGC27 cells. These results were further corroborated by the decreased tumour volume and weight, the reduced Ki67 positive cells, and augmented apoptotic cells in the *in vivo* assay. KLF4 was demonstrated to be an upstream effector of HADHB, and its expression was positively correlated with HADHB. KLF4 upregulation distinctly relieved the HADHB knockdown-induced enhancing effects on the viability, migration, and invasion of AGS cells. HADHB also regulating the Hippo-YAP pathway in HGC27 cells, which was confirmed by the pathway rescue assay. Collectively, these results indicate that the reduced expression of KLF4 induced the downregulation of HADHB in STAD. The reduced expression of HADHB triggered a series of tumour promoting effects by regulating the Hippo-YAP signalling pathway in STAD.

HADHB is a fatty acid beta-oxidation enzyme that was found to be downregulated in multiple cancers, such as OSCC, AML, and Wilms’ tumour (16, 17, 39). Previously, HADHB expression was found to be positively correlated with the OS of clear cell renal cell carcinoma (ccRCC) patients (40). The expression of HADHB was also found to be low in CRC tissues, and its overexpression notably reduced the migration and invasion of CRC cells (15). In this study, HADHB was found to be downregulated in STAD tissues, and its low expression was correlated with the poor OS of STAD patients. HADHB upregulation also suppressed various tumour cell phenotypes, such as cell viability, proliferation, and migration. These results aligned with those of previous investigations, and indicate that HADHB acts as a tumour suppressor gene in STAD.

KLF4, a functional TF with zinc-finger structure, is a member of the SP/KLF family. Several studies revealed the crucial roles of KLF4 in modulating cell differentiation, proliferation, apoptosis, and migration (41, 42). KLF4 is also considered a tumour suppressor gene in various cancers (43–46). Chen et al. revealed that KLF4 expression in GC tissues was extremely low, and the upregulation of its expression significantly repressed the viability, invasion, and migration of GC cells (47). The downregulation of KLF4 in STAD was also reported in another study, and high expression of KLF4 was found to be associated with a favourable OS of patients (48). KLF4 overexpression was verified to result in the inhibition of tumour cell migration and invasion by reducing the levels of metastasis-related MMP2, MMP9, and N-cadherin (49). In this study, KLF4 was found to regulate HADHB expression by directly binding to the promoter of HADHB, and its expression was positively correlated with the OS of STAD patients. The upregulation of KLF4 also notably abolished the HADHB knockdown-induced tumour promoting effects on cell viability, migration, and invasion. Of note, these results aligned well with those of earlier investigations. Collectively, these findings indicate that KLF4 might play a tumour suppressing role in STAD by regulating HADHB expression and modulating its downstream gene expression, such as proliferation- or metastasis-associated genes.

The Hippo-YAP signalling pathway is an evolutionally conserved pathway that is associated with tumour development (50–54). YAP is a downstream effector of this pathway (55). Several investigations revealed the crucial role of YAP in the regulation of cell apoptosis, proliferation, migration, and invasion (35, 38, 56). For instance, activated YAP was demonstrated to enhance cell proliferation and growth, and repress apoptosis (57). In contrast, silencing the expression of YAP suppressed cell proliferation, invasion, and migration (58, 59). YAP expression was previously confirmed to be increased in gastric adenocarcinoma (59). In the current study, HADHB upregulation markedly lowered the levels of YAP and TEAD4 in HGC27 cells. Further, YAP upregulation distinctly abolished the HADHB upregulation-induced repressing effects on the proliferation, migration, and invasion of HGC27 cells. These results align with those of earlier investigations. Collectively, these results indicate that HADHB upregulation led to tumour suppressive effects in STAD cells by regulating the Hippo-YAP signalling pathway.

Taken together, this study proved that KLF4 could directly bind to the promoter of HADHB to modulate its expression. HADHB upregulation may also suppress the viability, proliferation, migration, and invasion of HGC27 and AGS cells by regulating the Hippo-YAP pathway. These findings could provide a theoretical basis for the identification of prognostic biomarkers and targets for the treatment of STAD.

Taken together, this study proved that KLF4 could directly bind to the promoter of HADHB to modulate its expression. HADHB upregulation may also suppress the viability, proliferation, migration, and invasion of HGC27 and AGS cells by regulating the Hippo-YAP pathway. These findings could provide a theoretical basis for the identification of prognostic biomarkers and targets for the treatment of STAD.

## Data availability statement

The original contributions presented in the study are included in the article/supplementary materials. Further inquiries can be directed to the corresponding author.

## Ethics statement

The studies involving human participants were reviewed and approved by This study was approved by the Ethics Committee of The First Affiliated Hospital of Nanchang University (ID: IIT [2021] LLS No. 009, Nanchang, China). The patients/participants provided their written informed consent to participate in this study. The animal study was reviewed and approved by The animal experimental protocol was performed in accordance with the Guide for the Care and Use of Laboratory Animals and was approved by The First Affiliated Hospital of Nanchang University (ID: SD [2021] DLS No. 011).

## Author contributions

HX and YL conceived and designed the project. YL, J-BX and Z-GJ collected the data. HX and YL analyzed and interpreted the data. YL wrote the paper. All authors contributed to the article and approved the submitted version.

## Funding

The study was funded by Science and technology research project of Education Department of Jiangxi Province (GJJ200153); Research and Cultivation Fund for Young Talents, The First Affiliated Hospital of Nanchang University (YFYPY202104); Science and Technology Plan Project, Health Commission of Jiangxi Province (202310017); National Natural Science Foundation of China (no. 81960503).

## Conflict of interest

The authors declare that the research was conducted in the absence of any commercial or financial relationships that could be construed as a potential conflict of interest.

## Publisher’s note

All claims expressed in this article are solely those of the authors and do not necessarily represent those of their affiliated organizations, or those of the publisher, the editors and the reviewers. Any product that may be evaluated in this article, or claim that may be made by its manufacturer, is not guaranteed or endorsed by the publisher.

## Abbreviations

AML, Acute myeloid leukaemia; CRC, colorectal cancer; HADHB, dehydrogenase trifunctional multienzyme complex subunit beta; GC, Gastric cancer; MTP, mitochondrial trifunctional protein; OS, overall survival; OSCC, oral squamous cell carcinoma; STAD, Stomach adenocarcinoma.
